# Knowledge, attitude and practice towards medicines among school teachers in Lalitpur district, Nepal before and after an educational intervention

**DOI:** 10.1186/1471-2458-13-652

**Published:** 2013-07-13

**Authors:** Nisha Jha, Omi Bajracharya, P Ravi Shankar

**Affiliations:** 1 Department of Clinical Pharmacology & Therapeutics Department of Clinical Pharmacology & Therapeutics, KIST Medical College, Imadol, Nepal; 2 KIST Medical College, Imadol, Nepal; 3 Department of Clinical Pharmacology & Therapeutics, KIST Medical College, Imadol, Nepal

**Keywords:** Attitude, Children, Knowledge, Medicines, Nepal, Practice, School

## Abstract

**Background:**

Few studies regarding Knowledge, Attitude and Practice (KAP) towards medicines among school teachers have been carried out in Nepal. Obtaining baseline KAP is important to note deficiencies and plan appropriate interventions. School teachers have to know about medicines as they can be an important source of information about rational and safe use of medicines. The department of Clinical Pharmacology, KIST Medical College, Lalitpur, conducted a study regarding KAP of school teachers about medicines before and after an educational intervention from April 2011 to December 2011.

**Methods:**

The study was done in selected schools of Lalitpur district. Teachers were selected on a voluntary basis after obtaining written informed consent. Gender, ethnic or caste group, native place, age, educational qualifications, subject taught were noted. An educational intervention using a combination of methods like presentations, brainstorming sessions, interactive discussions using posters and distribution of information leaflets about the use of medicines was conducted. The KAP and overall scores among subgroups according to gender, age, level of education, subject, ethnicity, type of school (primary vs. secondary and government vs. private school) were studied. KAP and overall scores before and after the intervention was compared using Wilcoxon signed ranks test as the scores were not normally distributed.

**Results:**

A total of 393 teachers participated before and after the intervention. The median (interquartile range) knowledge, attitude and practice scores before the intervention were 63 (10), 23 (5) and 270 (48) respectively while the overall score was 356. The median knowledge, attitude and practice scores after the intervention were 71 (10), 28 (5) and 270 (48) respectively while the overall score increased to 369. Maximum possible score of knowledge, attitude and practice were 100, 40 and 320 respectively. Scores improved significantly for knowledge (p<0.001), attitude (p<0.001) and total scores (p<0.001) but not for practice (p=0.528).

**Conclusion:**

The intervention was effective in improving knowledge and attitude of the teachers. More studies among school teachers about their knowledge, attitude and practice about medicines are required in Nepal.

## Background

Almost half of all medicines globally are used irrationally. This can have severe consequences: adverse drug reactions, drug resistance, protracted illness and even death [1]. In addition, the financial cost incurred by individuals and governments due to irrational use is unnecessary and often extremely high, particularly in developing countries where patients often pay for medicines out of their own pockets [1]. Irrational use of medicines includes over-treatment of a mild illness, inadequate treatment of a serious illness, misuse of anti-infective drugs, over-use of injections, self-medication of prescription drugs and premature interruption of treatment. Data from many countries shows that such practices are frequent, and not exclusive to developing countries [1-3].

According to figures gathered by surveys presented to the World Health Organization (WHO) in 2000, about 60% of antibiotics in Nigeria were prescribed unnecessarily. In Nepal, over 50% of antibiotics prescribed in 1996 were not needed and 40% of medicines expenditure in the same year was wasted due to inappropriate prescriptions. Globally, the figure for unwarranted antibiotic prescriptions stands at roughly 50% [1,2].

Schools are considered a second home in Nepal, where students spend a large percentage of their time during childhood and adolescence. These days, students spend about seven to nine hours five or six days a week at school. The first place of learning is considered to be the home, where one is born and grows up with ones parents. School is the second place where the child learns how to interact, behave with others and to give and accept respect; therefore it is called the second home. Schools are organizations designed to influence and promote cognitive development and behavioral change. Health education provided by teachers may have an immediate effect on students and continue to influence students’ health behaviors into adulthood.

Strong beliefs among children and adolescents in the curative power of medicines [4], their limited knowledge about prevention and the easy accessibility to household medicines raises serious concerns. Since students spend most of their time in school with their teachers, proper education to the school teachers on the use of medicines will lead students toward a more rational and safer use of medicines. Self-medication among adolescents has become a serious problem that plays an important role in irrational use of medication and tends to increase with the age of adolescents [5,6]. A study done in Kuwait among school teachers, found that teachers considered teaching their students about the proper use of medicines so important that they felt it should be included in the national curriculum of health education [6].

Authors have consulted various reports and studies done within Nepal and outside [7-10] for identifying the problem areas in medicine use. A study done in Greece indicated irrational use of antibiotics and a need of exploring issues related to their use. This study explored parents' attitudes which often contribute to inappropriate prescription of antibiotics [10]. Similarly, another study done in Poland indicated that useful educational tools implemented in primary and secondary schools could play an important role in reducing the burden of community-acquired infections. The resource improves knowledge about microorganisms, hygiene and antimicrobial agents, and can be targeted at pupils, teachers and parents [11]. A pre-post comparison of a community intervention in Nepal has also shown the importance and benefits of educating school teachers and their impact on students [12].

Students at schools are taught various subjects including health and environmental health and science from class eight till class ten. Various topics taught are the human body, health and nutrition, physical education, health and physical fitness, community health and personal sanitation. Students are also given an orientation about common diseases. The topics of disease causing ‘germs’ and medicines which can be used to treat these diseases are also introduced. The information level about these topics gradually increases as the students progress through high school. About four to six hours are devoted to these topics every week. Teachers who teach these subjects have at least a Bachelor degree in biology.

Providing knowledge regarding use of medicine to school teachers is an important component of the overall health care delivery system of any country [13]. In developing countries such as Nepal where infant and early childhood mortality is high its importance cannot be overemphasized. For this reason the knowledge, attitude and practice among school teachers regarding use of medicines in Nepal needs to be evaluated. School teachers can help in rational use of medicines if they have the relevant information which can be transferred to students and through them to parents and the community.

The authors planned to study the knowledge, attitude and practice (KAP) regarding medicines among school teachers which is lacking in the Nepalese context. The association of KAP scores with demographic factors would help to identify subsections of teachers who may have deficiencies in knowledge and may need extra educational programs. In Nepal, there are various ethnic groups whose members have a common heritage, often consisting of a common language, a common culture often including a shared religion. The caste group is a system of social stratification in which communities are defined by thousands of endogamous hereditary groups called jatis. The jatis are formally grouped by the Brahminical texts under four well known categories, Brahmans (priests), Kshatriyas (kings, warriors, law enforcers, administrators), Vaishyas (agriculturists, cattle-herders and traders), and Shudras (lower caste menials, artisans, labourers, craftsmen, service providers) [14]. People belonging to the lower castes are disadvantaged and may have lesser knowledge and teachers from these disadvantaged groups have lesser opportunities for education compared to their counterparts. These people come from rural areas and face more hurdles. Lalitpur is one of the seventy-five districts of Nepal. The district, with Patan as its district headquarters, covers an area of 385 km^2^ and has a population (2001) of 337,785. It is one of the three districts in the Kathmandu Valley, along with Kathmandu and Bhaktapur. Its population was 466,784 in the initial 2011 census tabulation. Lalitpur District has many famous schools, colleges and hotels [15].

In Nepal classes from 1–5 are considered as primary school. The main objective is to reach children to read, write and do arithmetic. Secondary education (classes 6–8), stresses personality development and trains the students for higher learning. Classes 9 and 10, upper secondary, serve as the stepping stone for the higher secondary level. Classes 8 to 10 are termed as high school. Classes 11 and 12 are regarded as higher secondary where students take up different streams of study. Science, humanities, and commerce are the main courses for higher secondary study. The private schools in general have better facilities, are better managed and show better performance in the school leaving certificate (SLC) examinations as compared to Government schools. Government schools get financial aid for running the educational programs and hence the school fees are lesser compared to the private ones [16].

Rationale of the study: Studies have revealed major misconceptions about and irrational use of medicines in different countries. Studies regarding the knowledge, attitude and practice (KAP) of medicines among school teachers are lacking in the Nepalese context. As teachers could play an important role in educating their students and are regarded as learned and respected persons in local communities it is important that they have proper knowledge about medicines. This knowledge can be transferred to their students, their parents and members of the community. Authors thought that it is important to obtain their baseline KAP so that strengths and deficiencies can be noted and appropriate educational interventions planned.

This study was carried out with the following objectives.

a. To obtain knowledge, attitude and practice about medicines among school teachers of selected schools in Lalitpur district.

b. To note the association of knowledge, attitude and practice with demographic and other characteristics of school teachers and

c. To compare KAP scores before and after an educational intervention.

## Method

### Study duration

April 2011 to December 2011.

### Study design

Prospective longitudinal.

### Ethical considerations

Teachers were informed about the aims and objectives of the study and invited to participate. Written informed consent was obtained from all participants. The study was approved by the Institutional Research Committee (IRC) of KIST Medical College. Teacher’s knowledge, attitude and practice regarding medicines were studied using a questionnaire. A quasi-experimental study design with a control group which did not undergo the educational intervention would have reduced bias and made the study more powerful. This was not possible in our study as the school managements involved wanted all their teachers to benefit from the educational intervention. They were not in favor of denying a group of teachers the benefits of the intervention. As the teachers selected from each institution were those interested and willing to participate we were not sure about matching the respondents of the test and control group with regard to demographic characteristics. The issue was discussed during the review by and presentations before the experts of the UGC and the final study design was accepted and approved. The IRC of KIST Medical College also felt providing the educational intervention to all those involved would lessen the associated ethical problems.

### Study population

Selected school teachers from both government and private schools in Lalitpur district, Nepal

### Sampling method

Teachers selected were from primary and secondary levels of various government and private schools. Data about the schools in Lalitpur district were collected from the Lalitpur district education office. After obtaining this data, schools were chosen on the basis of their location. There are about 324 private and 198 government schools in Lalitpur district. We selected 17 schools from Lalitpur district. Out of these schools 11 schools were from urban areas and 6 schools were from rural areas. All the 11 schools from urban sector were private schools and the six government schools were from rural area. The sampling frame corresponded to distribution of schools in the district. Since the number of teachers in these private schools was more in the population (Lalitpur district), more teachers were included in the study sample. Specific schools from urban and rural areas were selected using random sampling method, i.e., lottery method. Total number of school teachers in the schools at Lalitpur district is approximately 21150. Among this, we selected 393 school teachers for our study.

### Sample size calculation

For sample size calculation, we assumed that the knowledge should be about 50% in our population of school teachers. This has been tested by pilot test and also assumed from literature review.

Knowledge=50%, P=0.5, Q=1-P=0.5

$$N={{\mathrm{Z\alpha}}^{2}\mathrm{xPxQ}/\left(M.E.\right)}^{2}$$

Where Zα =1.96 from normal table, two tailed

P= Population proportion

M.E = Margin of error

Now, n = (1.96)^2^ x (0.5) x (0.5)/(0.05)^2^

= 384

Non response correction = 5%

Total Sample size with provision for drop outs from the study= 384 + 5% of 384 = 403

Since no teachers dropped out from the study as they were an easily traceable population and we selected only schools whose managements were supportive of the study the sample size should be adequate for the study purposes.

### Study instrument development

The main objective of the study was to include questions based on the methodology of KAP studies about rational use of medicines conducted in other countries. Unfortunately literature about KAP of medicines among school teachers was scanty. To accomplish this goal, various health promotion models like the health belief model developed by Becker in the 1970s and Theories of reasoned action and planned behaviour developed in the 1980s by Ajzen and Fishbein and others were studied and applied emphasizing people’s intentions to change their attitudes and beliefs [17]. The health belief model suggests that the likelihood of an individual taking action with regard to a health problem is based on their perceived susceptibility to a health problem, their belief about its potential serious consequences, their belief that a particular course of action will reduce the probable consequences and an analysis whether the benefits of taking action will outweigh the costs or consequences [18,19]. The effects of irrational use of medicines on individual health, health of their family members and the potential serious consequences were emphasized. With regard to medicines many of the consequences were long term and not always direct so it was a challenge addressing some of these. We emphasized the important role of school teachers in the community and their vital importance in improving community health and improving the use of medicines. Goodman and colleagues suggested a four-stage model of organizational change which is also applicable to health promotion [20]. Stage 1 is referred to as awareness raising and involves stimulating interest and raising awareness about the problem at a senior level and also exploring solutions. Our intervention mainly focused at this level and during the educational sessions senior management of the schools were also present. Stage 2 is adoption of the change, stage 3 is implementation while stage 4 is institutionalizing the change. These further stages can be taken up in future. The stages of change model was developed by Prochaska and DiClemente and as a person plans to change a behavior he moves through different stages termed as precontemplation, contemplation, preparation, action and maintenance [21]. Our intervention mainly focused on the first three stages and further interventions may be needed to take the intervention to the next stages.

Health literacy is equipping people with the capacity to meet their healthcare needs in a complex society. An individual with an adequate level of health literacy has the ability to address his/her own healthcare needs, those of the family and the community [22]. A recent integrated conceptual model of health literacy details competencies related to accessing, understanding, appraising and applying health information [23]. In our questionnaire and in the educational sessions we discussed sources of medicines information available to the public in Nepal, how to understand and appraise the information and how to communicate medicine information to students and the public. Teachers as a part of their profession and training are equipped with these skills which were further strengthened by the educational intervention with special emphasis on medicines.

Manuscripts and published papers describing similar research and methodological issues were also studied [7,9,10,12]. The questionnaire was developed after discussions and deliberations among the researchers. After finalizing the statements, they were further discussed with other faculty members of the same department for their valuable inputs. Inputs were also obtained from other researchers in the field and from referees selected by the University Grants Commission (UGC) during the presentations and deliberations for short listing of research proposals for funding and during the detailed presentation of the study after acceptance. The questionnaire was pilot tested. The questionnaire was originally developed in English and translated into Nepali for better understanding by target teachers from primary and secondary section of different schools. The translated statements were given to Nepali language subject teachers for language correction. The questionnaire used is shown as an Additional file 1.

### Pretesting of the questionnaire

The questionnaire was pretested for readability and ease of understanding among twenty-two teachers. These teachers taught different grades like eight, nine and ten. Their data was not used in the study. Face and content validation of the measuring instrument was done by analyzing the data obtained from these teachers and corrections done where required. Cronbach’s alpha was measured as an indication of the internal consistency of the questionnaire. The value was 0.76 indicating good internal consistency.

### Demographics

Basic personal information like gender, age, ethnic/caste group, subjects taught in school, educational qualification and whether the respondent was originally from a village or town was noted.

### Scoring system used

Knowledge and attitude were studied by noting respondent’s agreement with a set of 28 statements using a Likert-type scale. The scoring system used was: 5 = strongly agree with the statement, 4 = agree, 3 = neutral, 2 = disagree and 1 = strongly disagree with the statement. The scores were reversed for negative statements. There were twenty statements for assessing knowledge with a maximum possible score of 100 and eight statements for attitude with a maximum possible score of 40. The practice component was measured by noting how often during the past month the respondent carried out a set of activities. Not at all was given the score 40, 1–2 times was scored 30, 3–5 times as 20, and more than 6 times as 10. The maximum score for practice part was 320. The practice pattern covered areas like the frequency of use of medicines like antibiotics, vitamins and tonics, checking expiry dates before purchasing medicine, giving medicines to students, taking a cough syrup, treating fellow staff members or giving advice about medicines. The total scores before and after the module was obtained by adding the ‘Knowledge’, ‘Attitude’ and ‘Practice’ scores. The maximum total score was 380. The median total ‘Knowledge’, ‘Attitude’, ‘Skills’, overall scores, and interquartile range were calculated. The questionnaire addressed different aspects about medicines like use of vitamins and tonics, deworming medicines, use of injections, cost of medicines, knowledge about expiry date, suspensions, controlled release preparations among others. The topics to be included in the questionnaire were developed on the basis of review of literature [7,8,12] and the authors’ experience of important medicine use problems in Nepal. Inputs were also obtained from other experts in the field.

To avoid bias certain statements were negative and their scores were reversed while calculating the total score. Knowledge, attitude and practice scores were compared before and after the intervention.

### Conduct of the study

School managements supportive of the study were selected and all interested teachers from primary and secondary levels in selected schools were involved in the research. The school management allotted a certain time period where the authors could interact with the teachers, administer the questionnaires and educate them about selected aspects of medicines.

This study was done in two phases. During the first phase the KAP of school teachers was studied before the educational intervention and the second phase was after intervention in the same group. The time interval between the first phase of the study and the intervention part of the study was fifteen days but was sometimes modified and delayed as per the convenience of the school management and availability of time for the researchers.

The intervention sessions included a training session, an hour long presentation on important issues related to medicines followed by a session using posters where we provided participants with the necessary information regarding medicines. The subject areas covered during the sessions were those where participants had less knowledge, poor attitude and/or practice as identified from responses to the questionnaire. The poster session focused on areas like antibiotic resistance, information about medicine labels and expiry dates. The prepared medicine information leaflets were distributed to all the study participants. The information leaflets dealt with reading label of a medicine, knowing about adverse drug reactions and side effects of drugs, appropriate use of antibiotics etc. Inputs about the process to be followed and topics to be discussed during the intervention process was obtained from medical educators in the institution, other researchers working in the field in the country, different health promotion theories [17,18], studies [7,19] and expert reviewers of the University Grants Commission who reviewed the study proposal and provided constructive comments. Two of the authors NJ and OB acted as facilitators for the intervention sessions with PRS who has advanced training in medical education providing critical inputs and support.

### Statistical analysis

After the intervention, the KAP scores were again measured using the same questionnaire. The collected data were analyzed using SPSS 17.0 for Windows. The knowledge, attitude and practice scores and total scores before and after the educational intervention were tested for normality of distribution using one sample Kolmogorov-Smirnov test. The samples were noted not to follow a normal distribution and median was calculated as a measure of central tendency, interquartile range as measure of variance and non-parametric tests were used for comparison between different subgroups of respondents pre-intervention. Scores before and after the intervention were compared using the Wilcoxon signed ranks test. Median knowledge, attitude, practice and total scores were compared among different subgroups of respondents with regard to variables like gender, age, ethnicity, educational qualification, government and private schools, subjects taught, and educational level being taught like primary and secondary level before and after the intervention using the Mann–Whitney test for dichotomous variables and Kruskal Wallis test when there was more than two subgroups of respondents. A p value less than 0.05 was taken as statistically significant.

## Results

### Respondent demographics

A total of 393 teachers participated before and after intervention. One hundred and ninety-one (50.1%) were male and 190 (49.9%) were female. There were 190 participants from the age group 21–30 years (49.9%) followed by 104 (27.3%) from the age group of 31–40. Two hundred and fifteen (57.5%) teachers were from primary level and 159 (42.5%) teachers were from secondary and higher secondary level. Majority of participants were Newars followed by Chettris. Most of the teachers were teaching Maths followed by English. Maximum teachers were holding bachelor degree followed by master’s degree. Two hundred and fifty-eight (65.6%) teachers were from private schools and 135 (34.4%) were from government schools. The total of different subgroups may not add up to 393 as certain respondents did not fill in all the required information. Table 1 shows the demographic characteristics of the respondents.

### KAP scores

The median (interquartile range) knowledge, attitude and practice scores before the intervention were 63 (10), 23 (5) and 270 (48) respectively while the median overall score was 356. The median (interquartile range) knowledge, attitude and practice scores after the intervention were 71 (10), 28 (5) and 270 (48) respectively while the overall median score increased to 369. Maximum possible score of knowledge, attitude and practice were 100 and 40 and 320 respectively. By using the Wilcoxon signed ranks test, knowledge (p<0.001), attitude (p<0.001) and total scores (p<0.001) increased after the intervention but the practice score did not improve (p=0.528).

The scores of certain statements for example ‘The term drugs and medicine are same’, ‘Injections cure diseases more quickly than orally taken medicines’, ‘Deworming tablets should be taken by chewing’, ‘Jeevan jal is not a powerful medicine as it is cheap’, ‘One drug can modify or alter the action of another drug’, ‘Medicines should not be used after their expiry date’, ‘All medicines cannot be used in case of pregnancy’ and ‘Suspensions need to be shaken well before use’ were low before intervention. These scores increased post-intervention (Table 2).

**Table 1:** Demographic characteristics of the respondents

**Characteristic**	**Number (percentage)** **n = 393**
Gender	
Male	191 (48.6)
Female	190 (48.3)
Not mentioned	12 (3.1)
Age	
Below 20	6 (1.5)
21-30	190 (48.3)
31-40	104 (26.5)
41-50	74 (18.8)
51-60	7 (1.8)
Above 60	3 (0.8)
Not mentioned	9 (2.3)
Teachers	
Primary	215 (54.7)
Secondary	159 (40.5)
Not mentioned	19 (4.8)
Ethnic/caste Group	
Brahmin	56 (14.2)
Chettri	106 (27.0)
Newar	145 (36.9)
Janajati	40 (10.2)
Not mentioned	46 (11.7)
Subjects	
Nepali	49 (12.5)
Science	45 (11.5)
Maths	105 (26.7)
English	80 (20.4)
Others	100 (25.4)
Not mentioned	14 (3.5)
Qualification	
Intermediate	20 (5.1)
Bachelors	225 (57.3)
Masters	126 (32.1)
Not mentioned	22 (5.6)
Type of school	
Private	258 (65.6)
Government	135 (34.4)

Table 2 compares the median ‘Knowledge’, ‘Attitude’, ‘Practice’ and total scores at the beginning and end of the intervention. The scores were significantly higher at the end of the intervention for knowledge, attitude and total scores.

**Table 2:** Median knowledge, attitude, practice and overall scores before and after intervention

**Characteristic**	**Median score**	**P-value**
Attitude		
Before Intervention	23	<0.001
After Intervention	28	
Knowledge		
Before Intervention	63	<0.001
After Intervention	71	
Practice		
Before Intervention	270	0.528
After Intervention	270	
Total score		
Before Intervention	354	<0.001
After Intervention	365	

The scores before and after the intervention were compared using Wilcoxon signed ranks test.

Table 3 compares the median total scores among various subgroups of respondents both before and after intervention.

**Table 3:** Comparison of median scores among different subgroups of respondents before and after intervention

**Characteristic**	**Knowledge**			**Attitude**			**Practice**			**Total**		
	**Before**	**After**	**P value**	**Before**	**After**	**P value**	**Before**	**After**	**P value**	**Before**	**After**	**P value**
Gender ^*^												
Male	63	70	<0.001	23	28	<0.001	262	270	0.378	352	365	<0.001
Female	63	72	0.001	23	28	<0.001	270	270	0.277	356	366	<0.001
Age (in years) ^Δ^												
Below 20	63	55.5	0.805	21.5	28.5	0.015	270	270	0.592	349	364	0.144
21–30	63	70	<0.001	23	29	<0.001	270	260	0.547	356	358	0.046
31–40	63	70	<0.001	23	27	<0.001	260	270	0.159	346	372	<0.001
41–50	61.5	71	<0.001	23	27	<0.001	270	280	0.006	356	378.5	<0.001
51-60	63	70	0.949	22	23	1.000	290	210	0.027	363	286	0.062
Teachers^ * ^												
Primary	63	72	<0.001	23	28	<0.001	270	270	0.440	351	366	<0.001
Secondary	63	72	<0.001	24	28	<0.001	270	270	0.423	350	365	<0.001
Ethnic/Caste group^Δ^												
Brahmin	63	71.5	<0.001	23	29	<0.001	260	270	0.457	348	372	0.004
Chettri	63	74	<0.001	23	28	<0.001	260	270	0.509	351	372	<0.001
Newar	63	68	<0.001	23	27	<0.001	210	270	0.309	353	363	0.030
Others	64	74	<0.001	23	28	<0.001	260	270	0.665	325	355	0.411
Subject taught^Δ^												
Nepali	63	70	<0.001	23	29	<0.001	270	270	0.457	355	372	0.031
Science	63	73	<0.001	23	29	<0.001	270	290	0.455	352	385	0.001
Math	63	72	<0.001	24	29	<0.001	270	270	0.576	357	365	0.211
English	63	67	<0.001	22	26.5	<0.001	270	270	0.945	356	356	0.224
Others	62.5	72	<0.001	22.5	27	<0.001	265	275	0.157	349.5	371	<0.001
Qualifications^Δ^												
Intermediate	62.5	69	0.223	23	23	0.017	255	270	0.895	337	346	0.845
Bachelor	63	70	<0.001	23	28	<0.001	270	270	0.281	353	362	<0.001
Master	62.5	71	<0.001	23	28.5	<0.001	270	270	0.452	359	371	<0.001
Type of school ^* ^												
Government	66	70	<0.004	22	28	<0.001	230	270	0.148	321	359	<0.001
Private	63	71	<0.001	23	28	<0.001	270	270	0.071	357	369	<0.001

* Mann–Whitney test was used as there were only two subgroups in this category.

Δ Kruskal Wallis test was used as there were more than two subgroups in this category.

### The scores of different subgroups of respondents before the intervention

### Knowledge

The scores for knowledge were same for both the male and female respondents. Similarly, the scores were same for all the age group respondents except the age group 41–50 years. The teachers from primary and secondary levels had the same knowledge scores. Teachers from other ethnic backgrounds had higher scores for knowledge. Teachers teaching different subjects were also having the same scores for knowledge whereas the scores were slightly less for teachers having intermediate and master’s degree qualification compared to those with a bachelor’s degree. Government school teachers were found to have higher scores compared to private school teachers.

### Attitude

The scores for attitude were the same for both genders. Teachers with age less than 20 years and in the age group 51–60 years showed lower scores compared to other age groups. Primary and secondary level teachers had the same attitude score. Teachers teaching different subjects showed variation in the scores. Teachers teaching English and subjects other than science, maths and Nepali had lower attitude scores. There was no difference in the scores for attitude among teachers according to qualifications. The attitude score was slightly higher among private school teachers.

### Practice

Practice scores revealed that females were having better scores as compared to males. Teachers from the age group 31–40 years had lower scores as compared to other age groups. Primary and secondary level teachers had the same scores while teachers from the Newar ethnic group had lower scores compared to other ethnic groups. Teachers teaching different subjects showed the same scores except teachers teaching other subjects whose score was 265. Government and private school teachers had the same scores.

### Comparison of scores before and after intervention of various subgroups of respondents

Table 3 mentions scores among subgroups of respondents both before and after the intervention.

### Knowledge

The knowledge scores improved significantly among females compared to males. The scores improved from 63 to 72. Similarly, the scores for knowledge were significantly improved for primary as well as secondary teachers after the intervention. Teachers from the caste group Chettris and other groups showed a significant improvement of knowledge after the educational intervention. Teachers with masters and bachelor level of qualification showed greater improvement in the knowledge scores compared to teachers having intermediate level of qualification. Private school teachers showed better scores than the government school teachers.

### Attitude

Males and females showed a significant change in their attitude about the use of medicines post intervention. All age group respondents showed an improvement in the scores which was statistically significant. Similarly, teachers from different ethnic backgrounds showed a significant improvement in the scores. Teachers teaching different subjects also showed similar type of improvement in the scores. Teachers having higher level of education showed more improvement in the attitude scores and both the government and private school teachers showed a significant improvement in scores.

### Practice

The increase compared to before the intervention was significant among the age group 41–50 years and 51–60 years. The increase if any among other subgroups of respondents was not statistically significant.

## Discussion

The overall median knowledge, attitude and total scores improved significantly post-intervention. Differences in KAP scores were noted among certain groups of respondents both before and after the intervention.

### Knowledge

The teachers who participated in our study represent different caste and ethnic groups of Nepal like Brahmins, Chettris, Newars, and Janajatis (Tharus, Limbus, Magars, Madhesis etc.) The median score for all castes improved after the intervention but significantly for chettris though Brahmins are still at the top of the caste hierarchy and have been considered to be highly intellectual since ancient times. Teachers coming from other ethnic groups are also called as Janajatis, who are the indigenous people of Nepal who have been traditionally oppressed and had low status in a caste dominated society. These teachers also have shown a significant improvement of knowledge. Recently seats have been reserved for them in educational institutions [14]. Scores also improved post-intervention according to educational qualification of teachers. This improvement was seen to be more significant in teachers with a higher level of education. Knowledge improvement has been shown to be associated with level of education in previous studies. A study done in Iran showed that the pregnant women’s knowledge about the use of folic acid and iron supplements had a significant relationship with the level of education [24]. In a recent study conducted among pharmacists regarding knowledge and attitudes toward herbal medicine, the median knowledge scores improved after the educational intervention [25].

Maximum number of schools in Lalitpur district is run by the private sector and comparatively less number of schools is run by the government. So more number of teachers were enrolled from private schools in our study. The result showed that there was improvement in the knowledge scores for private school teachers.

### Attitude

The attitude improved among males and female respondents. Similarly, teachers coming from all the ethnic backgrounds had significant improvement in the scores. This may be due to the changing scenario with people from different ethnic backgrounds now obtaining more facilities for education and growth. The median attitude and total scores improved after the intervention for all the age groups and teachers coming from different levels of government and private schools.

Attitudes or belief refers to traditional ideas, which are erroneous from an individual’s perspective, and this forms obstacles to appropriate behavior and treatment-seeking practices [26]. Attitude can be proper or improper. Proper attitudes can improve practice while improper attitudes can have a harmful effect. Measuring practice is a part of a standard KAP survey questionnaire. However, many KAP studies do not present results regarding attitudes and practices appropriately, probably because people may not express their real thoughts, beliefs and understandings which may be misinterpreted towards a particular subject area. This may have a direct impact on an individual’s thoughts [27].

### Practice

Practice has a direct impact on an individual’s behavior and change in practice can be obtained by carrying out more educational interventions for improving people’s practice.

Our study showed no improvement in the practice scores. A study done to evaluate knowledge, attitude and practice of self-medication among first year medical students in Bahrain showed that the practice for common over the counter medicines were not proper and were often inappropriate. This showed that even medical students do not have proper practice regarding safe use of medicines [28].

The reason behind this may be the duration of our educational intervention. Since, our intervention was for a short duration it may not be sufficient for substantial improvement in participant’s overall knowledge, attitude and practice level. Practice may be improved only after substantial changes in the knowledge and attitude level. Another problem about the practice part is the retention of the educational intervention provided to the participants. The educational intervention was conducted only once and was not repeated. The effect of the intervention on retention of information was not assessed. Lack of centers and information sources from where consumers can obtain unbiased, impartial knowledge about medicines may be another limitation which could have influenced practice.

### Queries from participants

Participants in the training session wanted more information about the duration and frequency of antibiotic use. They wanted detailed information about the use of herbal medicines and their safety profile. Some of the participants had a query about the beneficial and harmful effects after switching over to another brand of the same generic. Certain female participants were interested to know about the frequency of using anthelminthics for their child. Few questions were asked about the use of vitamins and tonics without any defined clinical indications. Vitamin supplements are commonly used with or without prescription by individuals for various reasons as an over the counter medicines. Certain participants did not know about the harmful effects of overuse of vitamins. This was very similar to a study done in Karachi, where knowledge, attitude and practice regarding the use of vitamin supplementation among patients visiting out-patient physicians were assessed was the most frequently raised question by the teachers. They were more concerned for the use of vitamins as a dietary supplement [29]. A similar type of study done at Nepal also revealed that the effect of intervention was associated with a significant increase in correct knowledge about action of antibiotics and excellent knowledge on methods of administration of antibiotics. The intervention was associated with a significant increase in knowledge on source of vitamins, and use of vitamin/tonics [12].

Dose of paracetamol and the use of its suspension was one of the commonest questions asked by female participants. Maximum number of participants agreed that they check the expiry dates prior to using a medicine and iron and folic acid should be given to pregnant women.

### Strength of the study

This study explores an area which has not previously been studied in Nepal to the best of our knowledge. It helps to understand the importance of knowledge transfer to the teachers and how the change in knowledge, attitude and practice can improve medicine use. This study enables lay persons to understand the importance of medicines like, antibiotics, deworming tablets, injections use and other important issues related to use of medicines.

### Recommendations

More studies are required so that detailed information about knowledge, attitude and practice are required. More resource intensive educational sessions are needed for better use of medicines by the consumers in a developing country like Nepal. Short training sessions or workshops should be arranged specifically for consumers like patients, school and college teachers to educate them about the safe and rational use of medicines. Further studies among a larger population of school teachers are required.

### Limitations

Our study had limitations. There was the problem of obtaining convenient time slots for conducting the study from school teachers and school management out of their busy schedule. Frequent shutdowns limited and hindered access to the study sites. Similarly, lack of free time for teachers apart from their normal duty hours was also a limitation. Besides, we could not gather all the teachers simultaneously for educational sessions due to various constraints. Certain schools were not responsive towards the study. We also did not employ a quasi-experimental study design employing a control group. The sample size was adequate but the number of teachers in certain subgroups was low.

## Conclusions

Knowledge, attitude and practice regarding medicines were noted among a sample of school teachers. Their association with demographics was measured. The scores were compared before and after the educational intervention. An educational intervention using different methods was found to be effective in significantly improving knowledge, attitude and total scores. Since, the intervention was carried out only once its effect on retention of knowledge was not measured. The practice scores were not significantly improved. More interventional research is needed on widely prescribed and utilized drugs and also on self-medication.

## Competing interests

The authors declare that they have no competing interests.

## Authors’ contributions

NJ was involved in conducting the study, conceptualizing the study, designing the questionnaire, collecting the data, reviewing the literature and writing the manuscript. OB helped in conducting the educational intervention, conceptualizing the study, designing the questionnaire, collecting the data, reviewing the literature performing the statistical analysis. PRS helped in conceptualizing the study, finalizing the methodology, performing statistical analysis and writing the manuscript. All authors have read and approved the final submitted version of the manuscript.

## Pre-publication history

The pre-publication history for this paper can be accessed here:

http://www.biomedcentral.com/1471-2458/13/652/prepub

## Supplementary Material

Additional file 1 Questionnaire for obtaining knowledge, attitude and practice towards medicines among school teachers in Nepal. Click here for file
