# Supplementary material for: Knowledge, attitude and practice towards medicines among school teachers in Lalitpur district, Nepal before and after an educational intervention
Source: BMC Public Health. 2013 Jul 13;13:652. doi: 10.1186/1471-2458-13-652 (PMC3744160; doi:10.1186/1471-2458-13-652)
Supplement: Additional file 1 — Questionnaire for obtaining knowledge, attitude and practice towards medicines among school teachers in Nepal. [file 1471-2458-13-652-S1.doc]

**Knowledge, attitude and practice towards medicines among school teachers in Nepal**

**Please answer all the questions including the basic details asked for**. Please do not write your name.

**Thank You very much for participating in the study! It is very much appreciated!**

**Gender: Age: Class teacher of ………… Ethnic/ caste group:**

**Subject taught: Educational qualifications: You originally belong to: Town/Village**

**For the following statements score using the following key (1 = strongly disagree with the statement, 2= disagree with the statement, 3= neutral, 4= agree with the statement, 5= strongly agrees with the statement.) Use whole numbers only.**

1. The terms drug and medicine are the same.
2. Medicines are always used on the prescription of a doctor.
3. All medicines can cause side effects.
4. One drug can modify or alter the action of another drug.
5. Injections cure disease more quickly than orally taken medicines.
6. Deworming tablets should be taken by chewing.
7. Medicines should not be used after their expiry date.
8. Iron and folic acid should be given to pregnant women.
9. All medicines cannot be used in case of pregnancy.
10. Vitamins and tonics make us stronger.
11. Digestive enzymes preparations are helpful in treatment of indigestion.
12. Suspensions need not be shaken well before use.
13. Jeevan Jal is not a powerful medicine as it is cheap.
14. Paracetamol in overdose is a powerful poison.
15. Narcotic drugs should be made freely available in medicine shops.
16. Controlled release preparations should be used without crushing the tablet/breaking the capsule.

1. Antibiotics should be taken for 5 days.
2. Antibiotics should not be used for treatment of common cold.
3. Herbal medicines are considered safe.
4. Medicines are not affected by storage conditions, like room temperature, moisture and direct sunlight.
5. Pain killers when taken on an empty stomach does not cause gastritis.
6. All cough syrups do not cause addiction.
7. Medicines can stimulate growth of hair on the head in bald men.
8. Medicines can improve memory and recall of information in school children.
9. Ayurvedic medicines and western medicines can be taken together with out problems.
10. Eye/Ear drops should not be used after one month of opening.
11. Liquid medicines should not be used when opened after one month.
12. Anti-hypertensive medicines are to taken for lifetime.

| S. No. | ***How often during the past month did you:*** | ***Not at all*** | ***1-2 times*** | ***3-5 times*** | ***6-7 times or more*** |
| --- | --- | --- | --- | --- | --- |
| 29 | Take any vitamins or tonics? |  |  |  |  |
| 30 | Use any antibiotics? |  |  |  |  |
| 31 | Develop an adverse effect from a medicine? |  |  |  |  |
| 32 | Buy medicines from medicine shop without a doctor’s prescription? |  |  |  |  |
| 33 | Check the expiry date prior to using a medicine? |  |  |  |  |
| 34 | Give any medicine to students in school? |  |  |  |  |
| 35 | Taken a cough syrup? |  |  |  |  |
| 36 | Have you treated a fellow staff member or given him/her advice about medicines? |  |  |  |  |
